# The complete chloroplast genome of *Scoparia dulcis* (Plantaginaceae)

**DOI:** 10.1080/23802359.2021.2011449

**Published:** 2021-12-23

**Authors:** Lin-Xuan Li, Yang Lin, Dan-Feng Tang, Chang-Qian Quan, Ying Liang, Shuang-Shuang Qin, Fan Wei, Kun-Hua Wei

**Affiliations:** Guangxi Key Laboratory of Medicinal Resources Protection and Genetic Improvement/Guangxi Engineering Research Center of TCM Resource Intelligent Creation, Guangxi Botanical Garden of Medicinal Plants, Nanning, PR China

**Keywords:** Complete chloroplast genome, illumina sequencing, phylogenetic analysis, *Scoparia dulcis*

## Abstract

Here, we report the complete chloroplast genome of *Scoparia dulcis* L. The genome is 153,701 bp in size. Two inverted repeats (IRs) with a total of 50,546 bp were identified. The rest of the sequence was separated into two single-copy regions, including a large single-copy (LSC) region (85,029 bp) and a small single-copy (SSC) region (18,126 bp), respectively. The genome of *S. dulcis* comprised of 129 genes, including 85 protein-coding genes, 8 rRNA genes, and 36 tRNA genes. Phylogenetic analysis showed that *S. dulcis* was strongly allied with *Bacopa monnieri.*

*Scoparia dulcis* L. is a perennial herb that belongs to the family Plantaginaceae, and it is distributed in low altitude areas of the tropics and subtropics. The chemical compounds isolated from this plant contain antiulcer, antidiabetic, antihyperlipidemic, anti-inflammatory properties and some other compounds were screened which have medicinal properties (Kota et al. 2019). There are many adulterants of *S. dulcis* on the market and it is difficult to distinguish them according to outward appearance, so it is a necessity to build up a method to differentiate it from other adulterating species. Chloroplast genomic information for *S. dulcis* will provide a candidate DNA molecular marker for the authentication of *S. dulcis* and identification of its adulterants. In this study, we sequenced, assembled, and annotated the complete chloroplast genome of *S. dulcis*, and analyzed its relationship with other related species by phylogenetic analysis.

The sample was collected from Qingxiu mountains, Guangxi province, China (108.417 E, 21.767N). The voucher specimens were deposited in Guangxi Botanical Garden of Medicinal Plants (http://www.gxyyzwy.com/, Fan Wei, email: fanwei_gx@163.com, under the voucher number GXNN-00782). Genomic DNA was extracted from silica-dried tender leaves using a modified CTAB method (Doyle and Doyle 1987). Whole-genome sequencing was conducted with the Illumina NovaSeq 6000 sequencing platform (Illumina, CA, USA). A total of 20,740,404 clean reads (150 bp paired-end read length) were used for the *de novo* assembly with NOVOPlasty using the chloroplast genome sequence of *Bacopa monnieri* (GenBank accession: NC 047469.1) as a reference sequence (Dierckxsens et al. 2017). Gene annotation was performed by the CPGAVAS (Liu et al. 2012), and the results were checked using DOGMA (Wyman et al. 2004).

The complete chloroplast genome of *S. dulcis* (GenBank accession No. MZ242235) with a size of 153,701 bp comprised of one large single-copy and one small single-copy region of 85,029 bp and 18,126 bp, respectively. This was separated by a pair of inverted repeat regions of 25,273 bp each. The overall GC content was 37.46%, while the corresponding values of the LSC, SSC, and IR regions were 35.39%, 31.49%, and 43.07%, respectively. A total of 129 genes were encoded, including 85 protein-coding genes (PCGs), 36 transfer RNA (tRNA) genes, and 8 ribosomal RNA (rRNA) genes. Among these, 18 genes were double copies and 18 unique genes (12 PCGs and 6 tRNA genes) contained intron, in which two PCGs (*clpP* and *ycf3*) had two introns while the others contained one intron.

To identify the phylogenetic position of *S. dulcis*, its chloroplast genome was aligned with 19 reported chloroplast genomes using MAFFT v.7.310 (Katoh and Standley 2013) and a maximum-likelihood (ML) phylogenetic tree was constructed using MEGA6.0 with Kimura 2-parameter as the nucleotide substitution model and 1000 bootstrap replicate (Tamura et al. 2013). As illustrated in Figure 1, *S. dulcis* appeared to be closely related to *Bacopa monnieri* with 100% bootstrap support. The complete chloroplast genome of *S. dulcis* will provide useful genetic information for further studies on species identification, molecular biology, evolution, population genetics, taxonomy or resources protection.

**Figure 1.**
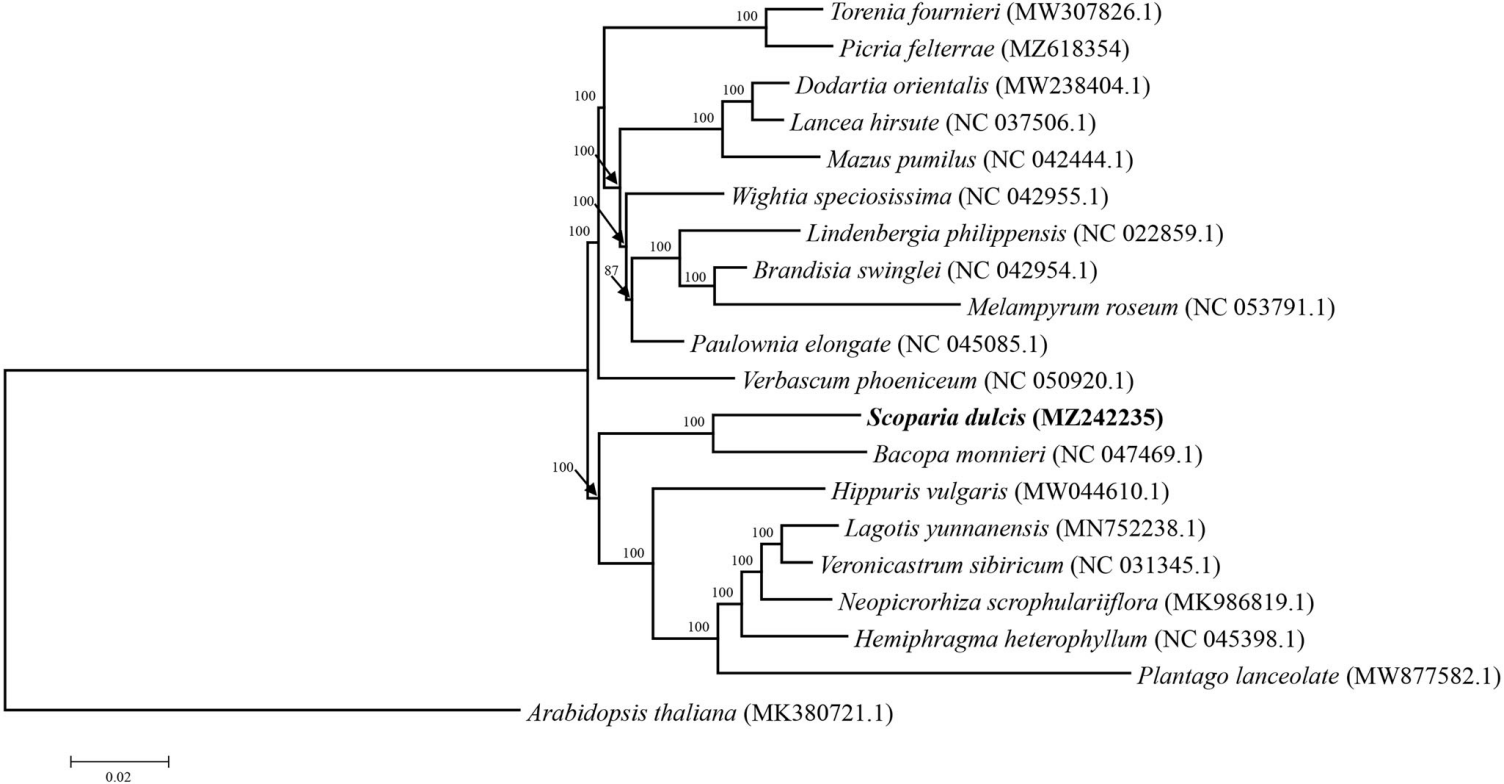
ML phylogenetic tree based on 19 species chloroplast genomes was constructed using MEGA6.0, Arabidopsis thaliana was used as the outgroup. Numbers on each node are bootstrap support values from 1000 replicates. GenBank accession numbers are shown in the figure.

## Data Availability

The data that support the findings of this study are openly available in GenBank at https://www.ncbi.nlm.nih.gov/genbank/, reference number: MZ242235. The associated Bio-Project, Bio-Sample and SRA, numbers are PRJNA739532, SAMN19791412 and SRR14883741 respectively.
